# Learning-by-Teaching Approach Improves Dengue Knowledge in Children and Parents

**DOI:** 10.4269/ajtmh.21-0253

**Published:** 2021-09-07

**Authors:** Maria Julia Hermida, Agustín Perez Santangelo, Cecilia Inés Calero, Carolina Goizueta, Manuel Espinosa, Mariano Sigman

**Affiliations:** ^1^ Instituto de Educación, Universidad Nacional de Hurlingham, Provincia de Buenos Aires, Argentina;; ^2^ Laboratorio de Neurociencia, Universidad Torcuato Di Tella, Ciudad Autónoma de Buenos Aires, Argentina;; ^3^ Consejo Nacional de Investigaciones Científicas y Técnicas, Ciudad Autónoma de Buenos Aires, Argentina;; ^4^ Instituto de Investigación en Ciencias de la Computación, Universidad de Buenos Aires, Consejo Nacional de Investigaciones Científicas y Técnicas Ciudad Autónoma de, Buenos Aires, Argentina;; ^5^ Área de Educación, Escuela de Gobierno, Universidad Torcuato Di Tella, Ciudad Autónoma de Buenos Aires, Argentina;; ^6^ Fundación Mundo Sano, Ciudad Autónoma de Buenos Aires, Argentina;; ^7^ Facultad de Lenguas y Educación, Universidad Nebrija, Madrid, Spain

## Abstract

There is narrow evidence on which strategies are most effective for disseminating information on dengue prevention. This is particularly relevant because social habits have a great prevention capacity for dengue. We investigated how effective are children as health educators, and how much they learn as they teach. We recruited 142 children and 97 parents in Argentina’s tropical area for two cluster randomized parallel trials. In Study 1, we compared the dynamics of dengue knowledge of 10-year-old children who—after receiving a dengue talk—1) listened to an unrelated topic; 2) read a booklet with information about dengue, 3) taught their parents about dengue, or 4) taught their parents about dengue, using the booklet. In Study 2, we assessed whether the parents’ dengue knowledge changed after interacting with their children, in comparison with parents learning about dengue from an expert or about an unrelated topic. Children that taught their parents what they learned, using a booklet, showed 2.53 more correct responses (95% CI [0.20, 4.85]; *P* = 0.027) than children who listened to an unrelated topic. This style of teaching also serves to effectively propagate knowledge: parents learned from their children the same as from an expert; and significantly more than parents who learned about an unrelated topic. Parents learned from their children even if they were taught with booklets (1.49, 95% CI [0.01, 2.96]; *P* = 0.048) or without (1.94, 95% CI [0.44, 3.44]; *P* = 0.006). Specifically, after being taught by their children, parents showed on average 1.49 (if they were taught with a booklet) and 1.94 (without booklet) more correct responses than parents that learned about an unrelated topic. The simple action of prompting children to teach consolidated their own knowledge and broadcasted it effectively to their parents. This strategy is a potential low to no-cost method for sharing information about dengue prevention.

## INTRODUCTION

Today, maybe more than ever, there is a general awareness of the importance of an effective education in public health issues.^1^ This is particularly relevant in the case of neglected tropical diseases, for which social habits have a great prevention capacity. Although historically unattended, these diseases have detrimental effects: according to the most recent estimations, today more than a billion people (one-sixth of the world’s population) are infected with one or more of them.^2^ Among neglected tropical diseases, dengue is paradigmatic example of a severe disease, that can only be prevented by community cooperation and in which health education is crucial for prevention.

Dengue is the most important vector-borne viral disease of humans, likely even more globally important than malaria in terms of morbidity and economic impact.^3^ It is also one of the fastest growing global infectious diseases, with 100–400 million new infections and 20,000 deaths every year, affecting most often poor populations.^4^ In 2019, there was an unprecedented increase in dengue epidemics worldwide and the WHO^5^ declared dengue virus as one of the top 10 public health threats, calling for governments, policy makers, and researchers to strengthen surveillance programs.

Currently, there is no cure for dengue infection. Therefore, the most effective actions involve prevention, and are directed to the control of* Aedes aegypti* mosquito, the virus’ vector.^6^ These actions include reducing the breeding sites, most of which are present in the domestic environment as a result of human action, and protection practices such as the use of repellents to prevent mosquitoes from biting.^7^ These prevention practices require healthy community habits, such as cleaning houses or eliminating breeding sites.^8^ But they also require permanent cooperation: one mosquito breeding site could start a disease propagation in the whole community.^6^ Health education and social mobilization are fundamental for the sustainable prevention^1^ and have been shown to be more effective than solely making new legislation to control dengue.^9^ Conveying a good understanding of dengue, its risks, and the benefits of healthy habits is thus at the core of the disease control.^10^

Schools are natural sources to disseminate this type of public health information.^9^^,^^11^^,^^12^ In countries where health education is not a curricular subject, it is generally carried out by experts from external agencies that provide public health talks to children;^12^ talks usually include booklets as support material, following the WHO recommendations.^8^ However, despite their wide and broad use, the impact of dengue health talks in schools is rarely quantified. Actually, we have scarce information on how best to conduct health education at schools,^11^ but also for general population.^13^

Here, we combine the basic research on core motivations of children learning inspired by cognitive neuroscience^14^ to examine how to make these health education interventions more effective. We applied the “learning by teaching” approach to dengue education, capitalizing on the natural leaning toward learning and cooperation.^15^^–^^18^ Specifically, we investigated how asking children to teach to their parents what they have learned can boost both their own and their parents’ knowledge. We built on two main ideas: first, humans teach instinctively,^15^^,^^19^ and while doing so they increase their own knowledge (as predicted by Seneca’s “docendo discimus,” when we teach, we learn).^20^^–^^22^ Secondly, children are natural teachers^15^^,^^23^ and they are able to communicate pedagogically information relevant to their tutees.^24^^,^^25^ Also, some studies showed visual information (e.g., written material, props, images, and knowledge maps) available at the moment of teaching promote learning,^26^^,^^27^ whereas some others showed that this material would decrease learning;^28^ thus, we analyzed how learning by teaching is modulated by visual material.

Learning by teaching literature is dominated by studies examining the learning benefits in the context of peer tutoring.^29^ However, the topics covered by that literature are mostly related to math and reading,^30^ but not to health education. Also, several studies have demonstrated that children can teach adults in a variety of issues as technology^31^ or climate change,^32^ but has been significantly less explored in health education issues.^33^^,^^34^ Here, we extend this idea to a stronger motivational core (children teaching their parents), and to an important, but unattended health issue (dengue disease).

To summarize, in Study 1, we investigated whether the exercise of teaching to their parents will improve children’s retention of the knowledge acquired during a dengue talk. We hypothesized that children who taught their parents what they had previously learned in the talk using visual support would increase and retain in the long term their own knowledge more than the other groups.

In Study 2, we analyzed whether this will convey an effective strategy to propagate relevant knowledge from children to parents. We hypothesized that scores from parents who learned from their children (with visual support) would not be significantly different than from those who learned from an expert. Additionally, to check that parents effectively learned from their children, we evaluated whether the number of dengue concepts mentioned by children was associated with parent’s dengue knowledge post interaction. We predicted that this correlation would be positive and significant in the group of parents who learned from their children with visual support.

## MATERIALS AND METHODS

### Participants.

In Study 1, we recruited 142 fourth graders (10 years old; 68 females, 1 unreported gender) and 57 parents (77% mothers, 13% fathers, and 10% caregivers). Parents gave their written consent (as well as children’s) to participate in the study.

Study 2 included 97 parents (85% mothers, 9% fathers, and 6% caregivers) of fourth grade children. Fifty-seven parents (the ones in Tutoring and Tutoring with booklet groups) were the same as in Study 1. Forty-one new parents were recruited and randomly assigned to two new groups: Unrelated Topic and Expert. They gave their written consent to participate.

Studies 1 (protocol n^o^ 683) and 2 (protocol n^o^ 435) were authorized by the Ethical Committee of the Centro de Educación Médica e Investigaciones Clínicas Norberto Quirno.

Participants’ recruitment and research activities were conducted in six suburban public schools from Puerto Iguazú (Argentina), a city endemic for dengue. Schools were predominately attended by low socioeconomic level population (59% of parents have low level of work, or they were directly unemployed). Evidence showed that low socioeconomic populations usually have less knowledge about dengue and showed fewer preventive practices; thus they are a main focus of dengue interventions.^35^ Also, according to schools’ authorities, children or parents had not received any dengue talk before.

### Instruments.

To evaluate dengue knowledge, we designed a 22-item True–False test about dengue (Supplemental Table 1, Supplemental Material). It was based on a questionnaire used in a previous study with similar population,^36^ but it was adapted to this sample by an interdisciplinary team of experts in mosquito-borne diseases and in children’s learning. The test evaluated the following contents: mosquito (i.e., physical appearance, life cycle, places where it lives, and moments in which it bites); prevention (i.e., actions to control breeding sites and bites); transmission (i.e., how it spreads); and symptoms (i.e., symptoms and actions that should be taken when symptoms appear).

Because we wanted to avoid as much as possible the learning effect produced just by the repetition of the same test, we designed three versions of the same True–False test. All versions have the same items but paraphrased and in random order. The number of true and false correct answers was similar (12 true, 10 false) in all versions.

Children (in Study 1) and parents (in Study 2) were randomly given one of the three versions of the True–False test, a pen and 10 minutes to answer the test. For each participant, the version given at each time was different. Importantly, no feedback about answers was given.

### Procedure.

#### Study 1.

We conducted a clustered randomized parallel trial (intended allocation ratio 1:1) to assess whether the learning by teaching approach increased dengue knowledge in children (acquired in a school talk).

First, children were evaluated for their level of dengue knowledge before the talk (baseline).

After that, all children received the talk from an expert (a health educator with 2 years of experience giving dengue talks in schools). It lasted about half an hour (X = 00:32:47, SD = 00:10:04) and included mainly theoretical content regarding the mosquito and dengue prevention, transmission, and symptoms. Also, a mosquito larva (inside a test tube), big pictures of mosquito, and drawings of dengue symptoms were shown. The talk had an interactive format: children were encouraged to ask questions during the lecture. The teacher officially in charge of each classroom as well as two assistants of researchers were present during the talk. Researcher’ assistants oversaw that all information that was asked in baseline-T3 was mentioned in the talk. If the expert forgot some content, assistants would remind her to say it, to make sure the talks were consistent across schools and that the 22 concepts about dengue evaluated in the outcome were given in the talk.

When the talk finished, children were evaluated again (T1) and were subsequently assigned to one of four groups:

Control (C): Children stayed in the classroom talking about an unrelated topic (instruction: “Tell me what you have been doing in school last week”; researcher’s assistants conducted the discussion in the whole group to any other topic than dengue)
Booklet (B): Children stayed in the classroom reading a dengue booklet individually (instruction: “Here you have a booklet, let’s read it!”)
Tutoring (T): In a silent part of the school, children taught their parents what they had learned in the talk (instruction: “Have you seen that you’ve learned a lot about dengue from the talk? Your mother/father wasn’t in the talk, so we thought you could explain to her/him what you have learned. Will you help us?”)
Tutoring with booklet (TB): In a silent part of the school, children taught their parents what they have learned in the talk, and a booklet was provided (the same instruction as for T, plus the following indication: “Here you a have a booklet for you to use in the explanation”).

All groups executed the activities, simultaneously, for 15 minutes approximately, in different places of the school. After completing the assigned activity, children were evaluated once again (T2). Baseline, T1, and T2 evaluations were taken on the same day. After T2, all children received the booklet. Finally, one month later, children were evaluated a last time (T3).

In order not to disturb educative activities, one of the groups in which children would teach parents (T or TB) and one of the groups where they would not interact with their parents (C or B) were randomly assigned to each school. Assignments of groups to schools were made by researchers before contacting schools’ authorities. Inside each school, children whose parents did not come to school, were assigned to C or B; and children whose parents came to school were allocated in T or TB. Because we did not find differences in the baseline among schools or between groups, data from all children were analyzed together. No school or child was aware of their intervention assignment.

#### Study 2.

In a clustered randomized parallel trial (intended allocation ratio 1:1), we assessed knowledge about dengue in parents who learned from their children (with and without visual support), in comparison with parents learning from an expert or about an unrelated topic.

First, parents were evaluated in their baseline level of dengue knowledge (baseline). After that, they were assigned to one of the following groups:

T: Parents were taught by their children. Children taught what they had learned in the talk.
TB: Parents were taught by their children what children had learned in the talk and a booklet was provided.
UT: Parents received an intestinal parasites talk from an expert.
E: Parents received a dengue talk from an expert.

Verbal interactions between children and parents in group T and TB were audio recorded (Mp3 format). To measure the number of concepts that were mentioned by the child during the parent–child interaction, these audios were coded by an assistant (blind to the study hypotheses) who assigned one point per each of the 22 dengue concepts mentioned (the 22 dengue concepts evaluated were the same 22 concepts of the outcome).

The dengue and intestinal parasites talks were given simultaneously, in different classrooms of each school, and lasted the same time. They had the same structure: definition of the vector (mosquito or parasite), contagion, symptoms, and prevention of infection.

After receiving the talks or interaction with children, parents were evaluated one more time (post score) on their dengue knowledge. All activities were carried out in the same day, one following the other, in different rooms of the school.

Assignments to T or TB groups followed Study 1 procedures; assignment to U and E group was made by researchers (shuffling names) before talks. No parent was aware of intervention assignment.

### Data analysis.

All analyses were conducted in R software^37^^,^^38^ considering α = 0.05 as the level of statistical significance. Raw data for all participants, which includes anonymized data collected during trials and analytic code, are available in https://osf.io/a629y/?view_only=dc47a00275aa4c1ebe7789ce1c325654.

We calculated the minimum sample size to achieve a power level of at least 0.80 using simulations (*simr* package) in Study 1; and Cohen’s method in Study 2. The minimum sample sizes were *N* = 120 and *N* = 84, respectively.

To ensure a basic test comprehension and motivation to complete it, we excluded data points with less than a 33% of correct responses. A data point is the score obtained by one subject, in one study group, at each time point (baseline, T1, T2, or T3).

In Study 1, to test the learning by teaching effect, we compared performance by group in time using a Linear Mixed Model with a baseline covariate. The dependent variable was Score. We included our two factors of interest (time and group), their interaction, and the baseline measurements as fixed effects factors into the model. Case (i.e., subjects’ ID) was included as a random effects factor for the intercept.

In Study 2, to test whether parents can learn about dengue from their children, we compared parents’ post score by group using a Linear Model with a baseline covariate. The dependent variable was *post* s*core*. We included our factor of interest (*group*), and the baseline measurements as fixed effects factors into the model. *Case* (i.e., subjects’ ID) was included as a random effects factor for the intercept.

Finally, to ensure that post score in parents was indeed reflecting what they had learned from their children, we ran a Linear Model with a baseline covariate; the dependent variable was mention.

Final models and other details are in Supplemental Material.

## RESULTS

### Study 1.

Test score means and dispersions for each time and group (Table 1) indicated that the difficulty of the test was adequate for children (there was no floor neither roof effect).

**Table 1:** Baseline and score descriptive statistics by group and time

	Baseline		T1		T2		T3	
	*n*	Mean (SD)	*n*	Mean (SD)	*n*	Mean (SD)	*n*	Mean (SD)
Control	39	10.15 (3.81)	36	15 (3.31)	26	14.31 (3.81)	22	13.64 (3.53)
Booklet	42	11.57 (4.07)	40	15.95 (3.66)	40	16.48 (3.18)	35	14.46 (3.24)
Tutoring	34	11.68 (4.81)	27	16.11 (3.53)	32	16.06 (3.42)	30	15 (3.55)
Tutoring with booklet	27	8.93 (4.74)	26	15.54 (3.99)	27	15.93 (3.99)	16	15.19 (2.97)

**Figure 1.**
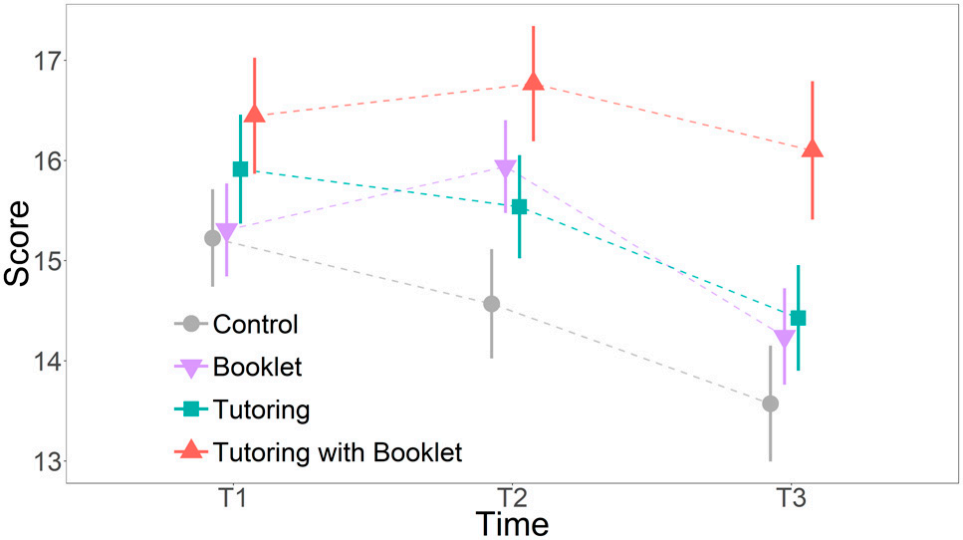
Score by group and time for children. Estimated marginal means for Score are represented with different shapes for each group and dashed lines join the marginal means by time within each group. Bars represent standard errors (SEM). Statistically significant differences were found between C and TB at T2 (*P* = 0.029) and at T3 (*P* = 0.027). This figure appears in color at www.ajtmh.org.

The linear mixed model revealed that the group TB had, on average, significantly higher scores in T2 and T3 than the control group (Figure 1, Table 2).

**Table 2:** Study 1: Linear mixed model

Contrast	Time	Estimate	SE	*df*	Lower CL	Upper CL	T ratio	*P* value
Booklet—control	T1	0.080	0.674	239.421	−1.664	1.825	0.119	0.999
Tutoring—control	T1	0.687	0.730	254.426	−1.202	2.576	0.941	0.783
Tutoring—booklet	T1	0.607	0.712	258.937	−1.234	2.448	0.852	0.829
Tutoring with booklet—control	T1	1.220	0.754	238.114	−0.730	3.171	1.619	0.370
Tutoring with booklet—booklet	T1	1.140	0.751	240.593	−0.803	3.083	1.518	0.428
Tutoring with booklet—tutoring	T1	0.533	0.799	250.776	−1.533	2.600	0.667	0.909
Booklet—control	T2	1.370	0.718	269.655	−0.487	3.226	1.907	0.227
Tutoring—control	T2	0.969	0.753	267.149	−0.977	2.915	1.287	0.572
Tutoring—booklet	T2	−0.401	0.689	241.569	−2.184	1.382	−0.582	0.938
Tutoring with booklet—control	T2	2.199	0.788	259.706	0.160	4.238	2.789	**0.029**
Tutoring with booklet—booklet	T2	0.829	0.748	238.415	−1.106	2.764	1.108	0.685
Tutoring with booklet—tutoring	T2	1.230	0.780	237.897	−0.789	3.249	1.576	0.394
Booklet—control	T3	0.669	0.753	291.335	−1.277	2.614	0.888	0.811
Tutoring—control	T3	0.855	0.783	285.602	−1.170	2.879	1.091	0.695
Tutoring—booklet	T3	0.186	0.710	258.754	−1.651	2.023	0.262	0.994
Tutoring with booklet—control	T3	2.526	0.900	313.917	0.201	4.851	2.806	**0.027**
Tutoring with booklet—booklet	T3	1.857	0.847	298.093	−0.330	4.045	2.194	0.127
Tutoring with booklet—tutoring	T3	1.672	0.879	295.129	−0.598	3.942	1.903	0.229

Comparisons between groups (expected values). *df* = degrees of freedom; SE = standard error. Significant differences (*P* value < 0.05, in bold) were found only between groups C and TB, and in timesT2 and T3. Estimates indicate the number of correct responses to which those differences are equivalent (e.g., in T3, TB group has 2.526 more correct responses than C group).

Specifically, at T2, children in the TB group (i.e., after interacting with their parents) had an average of 2.199 (95% CI [0.16, 4.24]; *P* = 0.029) more correct answers than children in the C group. No significant differences were found between the other groups in T2. One month after the intervention (T3), the TB group still showed 2.526 more correct responses (95% CI [0.20, 4.85]; *P* = 0.027) than the C group. Again, no significant differences were found between the other groups in T3. In sum, comparisons between groups indicated only two significant differences, both showing higher scores for TB compared with C group: TB had about two more correct responses than C in T2; in T3 that difference between groups increased to 2.5 more correct responses than C.

The final model’s *R*^2^ = 0.799. Additionally, we calculated the observed power for the difference between TB and C at T3 as a measure of reliability of our main finding. The estimated power was 81.80% (95% CI = [79.27, 84.15]). In line with this result, comparisons within groups showed that TB, between T1 and T3 (Supplemental Table 6, Supplemental Material), maintained the knowledge in the long term more than the others. Specifically, the TB group in T3 had 0.668 less correct answers than in T2, but that difference was not significant (95% CI [2.257, 0.921]; *P* = 0.583) and was the lower difference between T2 and T3 (C group, one month after T2 had 0.995 less correct answers; B had 1.696 less correct answers, and T had 1.110 less correct answers).

### Study 2.

**Figure 2.**
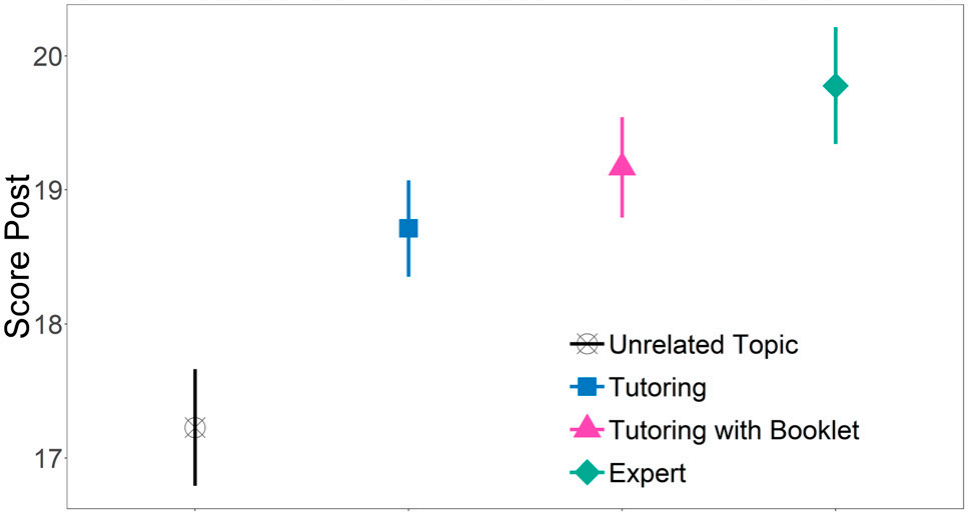
Score by group for parents. Estimated marginal means for Score Post are represented with different shapes for each group. Bars represent standard errors (SEM). Statistically-significant differences were found between UT and T (*P* = 0.048), UT and TB (*P* = 0.006), and UT and E (*P* < 0.001). This figure appears in color at www.ajtmh.org.

The results showed that the T, TB, and E groups had comparable post score, since no significant differences were found between the three groups (Figure 2, Table 3).

**Table 3:** Study 2: Linear model. Comparisons between groups (expected values)

Contrast (*df* = 92)	Estimate	SE	Lower CL	Upper CL	T ratio	*P* value
Tutoring—unrelated topic	1.485	0.565	0.007	2.962	2.63	**0.048**
Tutoring with booklet—unrelated topic	1.940	0.575	0.435	3.444	3.374	**0.006**
Tutoring with booklet—tutoring	0.455	0.519	−0.902	1.813	0.878	0.816
Expert—unrelated topic	2.550	0.616	0.937	4.163	4.138	**0.000**
Expert—tutoring	1.065	0.565	−0.412	2.543	1.887	0.241
Expert—tutoring with booklet	0.610	0.575	−0.894	2.115	1.061	0.714

*df* = degrees of freedom; SE = standard error. *P* value < 0.05 indicate significant differences (in bold). Estimates indicate the number of correct responses to which those differences are equivalent.

Parents learned from their children the same as from an expert, even if the children used a booklet (0.61, 95% CI [−0.89, 2.12]; *P* = 0.714) or not (1.07, 95% CI [−0.41, 2.54]; *P* = 0.241). However, groups E, TB, and T had significantly higher post score than UT group. Specifically, compared with UT, T had an average of 1.49 (95% CI [0.01, 2.96]; *P* = 0.048) more correct answers, TB had 1.94 (95% CI [0.44, 3.44]; *P* = 0.006) more correct answers, and E group had 2.55 (95% CI [0.94, 4.16]; *P* = 0.000) more correct answers. The final model’s *R*^2^ = 0.320 and the observed statistical power was 99%.

Finally, we tested whether the number of dengue concepts mentioned by children during tutoring was associated with parents’ post score. Results (Supplemental Table 12, Supplemental Material) indicated that the number of mentions significantly predicted parents’ post score (β = 0.163; *t*_(53)_ = 2.313; *P* = 0.025). R-square value (*R*^2^ = 0.170) indicated that 17% of variance in post score was explained by baseline and mention.

## DISCUSSION

The demonstration that teaching to parents increases children’s long-term retention of dengue information as well as parent’s dengue knowledge is shown here for the first time to the best of our knowledge and presents an easy tool to structure long-lasting cognitive interventions in dengue. Study 1 demonstrated that asking children to teach what they learned, using a simple material support (a booklet) improves children’s knowledge about critical aspects of dengue. This improvement is observed in terms of quantity of information immediately after the intervention, but most importantly it also shows an effect in the long-term retention (even 1 month later). From a practical perspective, this turns out to be the most critical result, because interventions are only effective if the acquired knowledge is retained in time and does not fade out rapidly. In turn, this could increase children’s capability to spread the information during that time. Their capacity to do so is demonstrated in Study 2 where we showed that children are, as teachers, as effective as what is reached in an intervention targeted directly to adults. In sum, the learning by teaching strategy (usually used for other educative contents) could be effective for health education, particularly in the case of dengue that require cooperative prevention actions. Importantly, results demonstrated that it is not necessary to reach parent and children by doubling efforts (economic, social, and time) of the intervention. Instead, communicating to children in schools would suffice to effectively disseminate knowledge to their parents.

Importantly, findings from Study 2 raise the issue of understanding why in this setup children are as effective as experts. We suggest two distinct (but related) possibilities. The first one is that the specific dengue content that needs to be conveyed is relatively simple, saturates rapidly, and can be well understood by a child with comparable depth to an adult expert. This strategy would not be valid if the concepts involved would be much more difficult to grasp by children. Evidence in favor of this comes from the fact that parents’ learning varies with the quantity of dengue concepts expressed by children. A second important issue is the motivation factor and differential attention deployed by parents to their children, compared with a public expert. This will certainly be specific of this particular interaction and may not extend (although it should be tested) to random children teaching random adults, but it was precisely this link that was at the core of the rationale for using this strategy. Also, further research should explore individual differences in the effects on receptivity of parents as well as retention among children.

On the other hand, our results suggest that providing material as support for teaching is crucial to enable teacher’s learning, as others have previously suggested.^26^^,^^27^ Previous studies have reported the effectiveness of dengue booklets in changing knowledge and habits.^8^ Therefore, we expected a boost on the TB group and our results confirmed our hypothesis. This is opposite to Koh et al.,^28^ who found that teaching without material (analogue to our T group) and retrieving information without teaching it (analogue to B group) were more effective than teaching the material with notes (analogue to TB group). Besides differences in experimental settings (type of topic taught and its relevance, preparation to teach, and age of tutors and tutees), we cannot explain that retention as a memory retrieval effect. Instead, our results are in line with the idea that when tutors actually teach a content of knowledge, they may develop a more persistent understanding of the material,^39^ and that visual support is an information organizer,^40^ helping to monitor their own understanding and giving the possibility of recognize and repair knowledge gaps;^39^ the booklet could function as an assistant for their own learning process, especially considering that our children were not given time to prepare the tutoring.

Finally, other studies have shown improvements in parent’s knowledge through placing children in the role of their teachers regarding asthma,^33^ cardiovascular risks factors,^34^ or obesity.^41^ However, in the case of dengue, this is the first study showing that children improved parents’ knowledge (although others showed improvement in children’s,^36^^,^^42^ and, separately, in parents’ knowledge).^43^ A possible explanation for this is that most studies have assumed that health education flows primarily from parent to child.^33^ Our study goes on the opposite way, and demonstrated that children can be successful educators also in the case of dengue.

### Limitations.

Firstly, for our experiments we promoted a child–parent interaction that may not naturally occur in real life. However, people in charge of public health talks can easily promote this interaction asking children as homework to teach their parents. Secondly, the True–False test used was developed specifically for the studies and not previously validated. Nevertheless, it is a direct test of dengue knowledge, and showed no floor or roof effects. Also, almost all scores were higher than chance, showing that it was understood by the majority of participants. Thirdly, dengue is endemic in the region where our study was conducted^4^ and highly dangerous;^5^ thus, results could have a biased effect of relevancy (i.e., it could have made children and parents pay more attention, in comparison with a less urgent topic). Finally, although having knowledge is a prerequisite, it is not sufficient to change habits, and other dimensions are needed.^10^ Future studies should inquire whether the amplifying effects, we observed, can translate to habits change.

### Implications.

What do our results mean in terms of the cost–benefit balance of educational interventions? After teaching their parents using a booklet, the TB group showed approximately two more correct answers (i.e., dengue concepts) than children who only received the talk (control group, C); 1 month later, the TB group had 2.5 more correct answers than control.

To understand the magnitude of these differences, we put them in the context of results from similar studies. In comparison with a control group, putting flipcharts or posters in schools was associated with less than one more correct answer in a dengue questionnaire;^44^ a 2-month board game and a theoretical intervention were associated with three and two more correct answers than controls, respectively;^45^ and a 3-month educational intervention was associated with five more correct answers.^46^

Similar observations could be made for parents’ knowledge. Parents in our T and TB groups have 1.5, and 2 points more than controls (i.e., one and a half and two concepts). Other educative interventions that measure effects on parents’ knowledge showed mixed results (intervention increase parent’s knowledge about mosquito life cycle, but to decrease parent’s knowledge about dengue causes)^43^ or the acquisition of one concept.^47^

Considering these results, teaching parents with a booklet produces intermediate gains in children dengue knowledge, higher than,^44^ similar to,^45^ and lower than^46^ other elementary educational interventions. Also, regarding parents, this approach produces greater gains than other educational intervention. However, considering that teaching parents with a booklet is a low time-consuming intervention that takes less than 10 minutes, the time efficiency of the learning by teaching approach highlights its potential value. In other words, with a 10-minute intervention, we observed effects similar in magnitude to those with 2-month interventions.^45^

Besides that, it is important to consider monetary costs. According to our results, the action that greatly increased learning in children was teaching to their parents using a booklet. There are two monetary costs involved in this action: an expert giving a 45-minute talk in a school and one booklet per child. At the place where the study was conducted, the hourly rate of the dengue expert is 8.03 USD, including transport to school; and to print booklets for a class (typically, 25 children) has a cost of 1.3 USD. In sum, the total cost for reaching 25 children and 25 parents (in the TB group) was 9.33 USD in our study. Besides those costs, it is important to state that there already exist numerous programs sustained by the state or NGOs that are trying to reach children and adults through talks given by experts,^12^ and most of them include booklets.^8^ In fact, this is the typical dengue educational intervention.^48^ Thus, in comparison with these already existing interventions, the learning by teaching approach has no extra cost.

Finally, although educational interventions not necessarily translate into preventive actions,^48^ they are a central part of vector-control strategies^49^ and having knowledge is a prerequisite to social mobilization and habits change.^10^ Our findings only refer to this initial point of the vector-control process: increasing dengue knowledge. For this aim, it is a low-cost, short, and effective action that could increase the potential value of the expert talks.

## CONCLUSION

We found that children who taught their parents about dengue improved their own dengue knowledge, specifically when teaching with visual material. Also, parents learned from their children about dengue the same as from an expert.

Our findings showed that learning by teaching with booklets, generates long-term retention of dengue knowledge in 10-year-old children (something that just a unique dengue talk did not reach). Also, we showed that children can be as effective as an expert in teaching about dengue to their parents, demonstrating that it is not necessary to reach parent and children by doubling efforts (economic, social, and time) of giving talks. The simple suggestion (i.e., “teach what you have learn”) can generate a synergic win–win situation beneficial for children as well as for parents. We recommend continuing with dengue talks given by experts in schools, but after it giving children the explicit instruction of teaching their parents what they have learned in the talk, using a booklet. There are different easy pedagogical ways of encourage the children to became health educators (e.g., regular teachers can ask as homework to teach parents using the booklet and register the experience in a video, audio, or written format; or to share the “at home teaching experience” with the whole group). Based on our results, we could expect that these actions would increase children’s and parent’s dengue knowledge.

## Supplemental Material


Supplemental materials
