# Supplementary material for: Learning-by-Teaching Approach Improves Dengue Knowledge in Children and Parents
Source: Am J Trop Med Hyg. 2021 Sep 7;105(6):1536–43. doi: 10.4269/ajtmh.21-0253 (PMC8641307; doi:10.4269/ajtmh.21-0253)
Supplement: Supplementary file 1 [file tpmd210253.SD1.pdf]

Supplemental Material

Learning-by-teaching approach improves dengue knowledge in children and parents

Studies 1 and 2: Participants. Sample size calculation. For sample size calculation in Study 1, given the heterogeneity in cross-age tutoring studies' effect sizes,[1] we used the lowest (d= .38) and the highest (d= .80) reported effect sizes to derive a range of sample sizes required to achieve a power level of at least .80. The sample sizes required were n=540 and n=120, respectively. We aimed at recruiting the highest required sample size and therefore invited 4th grade children in all the sub-urban, low socioeconomic status schools in the city (potentially reaching 600 children). However, only 155 children participated in the study (the others did not sign their consents or were absent during the time in which activities were carried out in schools). For Study 2 we calculated the minimum sample size required for an anticipated effect size of .15, a statistical power level of .80, four factors and a probability level of .05 (see [2]). The minimum sample size required is 84.

Study 1: Data analysis. To test our hypotheses, we used a Linear Mixed Model with a baseline covariate since this statistical approach (aside from accounting for inter-subject variability via random effects factors) has the advantage of being able to deal with unbalanced data (which is the case in this study).

Following descriptive data exploration, we assumed normality of standardized residuals, homogeneity of variance between groups and normal distribution of random effects.

Although our statistical approach was mainly hypothesis-driven, we performed likelihood ratio tests between nested models including (or not) interaction terms and nuisance-variables (school ID, True-False test version, etc.) to select the best model (i.e., highest likelihood with the smaller number of parameters). Model diagnostics (residual plots and Shapiro Wilks and Levene tests) after estimation of the selected model, showed that LMM assumptions were not violated, thus providing credibility to model inference.

To validate the selected model, we visually inspected residuals (to check for obvious departures from randomness) and computed the R^2 of the relation between observed data and model predictions as a measure of goodness of fit. We then performed Tukey HSD posthoc tests (adjusted for multiple comparisons) over the estimated marginal means differences to address our hypotheses (i.e., differences for each time and group). Degrees of freedom were computed with the Kenward-Rogers' approximation for LMM.

We used specific R packages for each part of the analysis pipeline: tidyr[3] and dplyr [4] for data manipulation; lme4 [5] for linear mixed model estimation, car [6] for ANOVA, emmeans [7] for post-hoc hypothesis testing, flextable[8] for table output, and ggplot2 [9] for plotting.

The final Linear mixed model formulae and specifications for Study 1 is the following:

Random Intercepts Linear Mixed Model (LMM1):

$$Y_{ij} = \beta_0 + \beta_1 * BL_{ij} + \beta_2 * T_{2ij} + \beta_3 * T_{3ij} +$$
$$\beta_4 * G_{2ij} + \beta_5 * G_{3ij} + \beta_6 * G_{4ij} +$$
$$\beta_7 * BL_{ij} * T_{2ij} + \beta_8 * BL_{ij} * T_{3ij} +$$
$$\beta_9 * G_{2ij} * T_{2ij} + \beta_{10} * G_{3ij} * T_{2ij} +$$
$$\beta_{11} * G_{4ij} * T_{2ij} + \beta_{12} * G_{2ij} * T_{3ij} + \beta_{13} * G_{3ij} * T_{3ij} + \beta_{14} * G_{4ij} * T_{3ij}$$
$$+ \alpha_j + \varepsilon_{ij}$$

Y : Dependent variable (Score).

BL: Baseline co-variate.

$T_{2,3}$: Dummy-coded variables for Time 2 and 3.

$G_{2,3,4}$: Dummy-coded variables for Groups

- G_2: Booklet,
- G_3: Tutoring,
- G_4: Tutoring with booklet.

Note: T_1 and G_1 (Control) are the reference levels for $T_{2,3}$ and $G_{2,3,4}$, respectively.

$\alpha_j \sim N(0,\sigma^2_c)$, σ^2_c: cases variance.

$\varepsilon_{ij} \sim N(0,\sigma^2_r)$, σ^2_r: residuals variance.

i: 1-3 observations per case.

j: 1-155 cases.

Study 1: Results details.

Regarding model fitting information, the estimates for each level are in Table SM2, the intraclass correlation coefficient is in Table SM3, and the likelihood ratio test is in Table SM4. The final model's R^2= .799, indicating an adequate fit (i.e. the model fairly reproduces the observed data). Although baseline variability

was integrated into our model, we ran a separate analysis on baseline values only and found no differences between groups (Table SM5). Results of comparisons within groups are in Table SM6.

Study 1: comparisons within groups interpretation

Regarding how meaningful these within-groups differences are, between T2 and T3, only the B group decreased significantly (the others decreased but in a non-statistically significant amount). However, TB group decreased less than the others. The decrease in TB group was half of the decrease in C and T, and one third of B group. Differences between half a point and one and a half points are comparable to the ones that have been reported in other dengue educational interventions analyzing within differences. For example, AhbiRami and Zuharah [10] carried out an intervention in secondary schools to increase knowledge about dengue, and found significant differences between the pre and post-test within groups in 1 or 2 items (according to the participant's residence area). Also, in T3, only the TB group showed a level of knowledge similar to the reached after the talk (T1), suggesting that teaching parents with a booklet is meaningful to maintain knowledge in the long term, maybe working as a protection against the of lost dengue concepts acquired in the talk due to time.

Study 2: Data analysis.

We ran models in R (version using the same functions and packages as in Study 1).

Model selection, diagnostics and validation and hypothesis testing was performed in the same way as for Study 1 (except here there was not a random-effects factor). The final model is the following:

Linear Model (LM2):

$Y_i = \beta_0 + \beta_1 * BL_i + \beta_2 * G_{2i} + \beta_3 * G_{3i} + \beta_4 * G_{4i} + \varepsilon i$

Y : Dependent variable (post score).

BL: Baseline co-variate.

$G_{2,3,4}$: Dummy-coded variables for Groups

- G_2: Tutoring,
- G_3: Tutoring with Booklet,
- G_4: Expert.

Note: G1 (Unrelated Topic) is the reference level for $G_{2,3,4}$.

$\varepsilon i \sim N(0,\sigma^2_r)$, σ^2_r: residuals variance.

i: 1-96 cases.

Study 2: LM2 results details. Means and dispersions for each time and group are in Table SM7. Regarding model fitting information, the estimates for each level are in Table SM8. The final model's R^2= .320, indicating an adequate fit (Table SM9). No differences between groups were found in baseline (Table SM10).

Study 2: Linear model including mention (LM2m) results details.

Descriptive statistic of mention and post score, in T and TB groups is in Table SM11. Model estimation is in Table SM12 and fitting information and other data are in Table SM13. The final model's R^2= .170.

SM Tables

Table SM1. True-False Items in Test (Version A), and Correct Answer for Each Item

True-False item	Correct answer
There is more dengue in the jungle than in the city	F
Dengue is transmitted from human to human	F
The Aedes aegypti mosquito leaves its eggs in streams	F
Dengue, Zika, Yellow fever and Chikungunya fever are transmitted by all mosquitoes, infected or uninfected ones	F
Dengue is a disease that is never fatal	F
If you feel a fever or headache and think you may have dengue, you have to take an aspirin, it is not necessary to go to the doctor	F
The mosquito that transmits dengue, in general, bites at noon	F
If you feel a fever or headache, you have to stop drinking water	F
The mosquito, since it becomes an adult, can live for 4 to 8 weeks	T
Only the female mosquito transmits dengue	T
In order to avoid mosquitoes, you have to throw containers that collect stagnant water	T
Symptoms appear 4 to 7 days after the mosquitoe bites you	T
First, the mosquito is larva and lives in the water for about 7 days	T
The mosquito is found in dark places inside homes	T
Aedes aegypti is black and has white spots and stripes on the body and legs	T
If we have a container that collects stagnant water and we cannot throw away, we must cover it so that it does not become a mosquito breeding site	T
After a rainy day, you have to remove the containers that contain water to prevent the mosquito from growing	T
For the mosquito does not bite you, you have to wear short-sleeved clothes	F
The mosquito bites more frequently on the face and back, than on the legs and arms	F
The use of repellents can help avoid the mosquito bites you	T
In order to avoid mosquitoes you have to keep the house tidy and clean	T
Dengue causes fever and body aches	T

Table SM2. Study 1: LMM1 Coefficients Estimates

Coefficient	Estimate	SE	df	t value	Pr(>|t|)	2.5%	97.5%
Intercept	9.366	.807	253.164	11.603	.000	7.810	10.922
Baseline	.540	.061	257.965	8.784	.000	.422	.659
T2	.457	.836	222.469	.547	.585	-1.150	2.066
T3	-.183	.900	226.403	-.203	.839	-1.916	1.549
Booklet	.080	.674	238.050	.119	.905	-1.222	1.378
Tutoring	.687	.730	253.181	.941	.348	-.722	2.094
Tutoring with Booklet	1.220	.754	236.733	1.619	.107	-.234	2.674
Baseline : T2	-.103	.061	214.705	-1.682	.094	-.220	.015
Baseline : T3	-.135	.065	223.919	-2.074	.039	-.261	-.010
T2 : Booklet	1.290	.700	216.707	1.843	.067	-.057	2.636
T3 : Booklet	.588	.738	221.331	.798	.426	-.832	2.007
T2 : Tutoring	.282	.755	219.834	.373	.709	-1.171	1.736
T3 : Tutoring	.168	.788	223.419	.213	.832	-1.348	1.684
T2 : Tutoring with Booklet	.979	.770	216.412	1.271	.205	-.503	2.460
T3 : Tutoring with Booklet	1.306	.882	224.272	1.481	.140	-.390	3.003

Table SM3. Study 1: LMM1 Intra-class Correlation Coefficient

	Variance	ICC
Case	4.616	.538
Residual	3.957	

Table SM4. Study 1: Nested LMM Likelihood Ratio Test (Tests Random Effects Factor “Relevance/Significance”)

Df	Log Likelihood	Df	Chi squared	Pr(>Chi squared)
16.000	-881.084			
17.000	-843.602	1	74.965	.000

Table SM5. Study 1: ANOVA Between Groups in Baseline

Contrast (df=138)	Estimate	SE	Lower CL	Upper CL	T ratio	p value
Booklet - Control	1.418	.961	-1.082	3.917	1.475	.456
Tutoring - Control	1.523	1.014	-1.115	4.16	1.501	.44
Tutoring - Booklet	.105	.997	-2.488	2.698	.105	1.000
Tutoring with Booklet - Control	-1.228	1.082	-4.042	1.587	-1.135	.669
Tutoring with Booklet - Booklet	-2.646	1.066	-5.419	.128	-2.481	.067
Tutoring with Booklet - Tutoring	-2.751	1.114	-5.648	.147	-2.468	.069

Table SM6. Study 1: Linear Mixed Model. Comparisons Within Groups

Contrast	Group	Estimate	SE	df	Lower CL	Upper CL	T ratio	p value
T2 - T1	Control	-.657	.535	226.361	-1.918	.604	-1.229	.437
T3 - T1	Control	-1.652	.565	226.357	-2.984	-.320	-2.927	.011
T3 - T2	Control	-.995	.620	235.882	-2.459	.468	-1.604	.246
T2 - T1	Booklet	.633	.449	208.201	-.428	1.693	1.408	.339
T3 - T1	Booklet	-1.064	.475	218.584	-2.184	.057	-2.241	.067
T3 - T2	Booklet	-1.696	.475	218.557	-2.816	-.576	-3.574	.001
T2 - T1	Tutoring	-.375	.533	216.931	-1.633	.884	-.703	.762
T3 - T1	Tutoring	-1.485	.550	223.453	-2.783	-.186	-2.698	.020
T3 - T2	Tutoring	-1.110	.516	213.854	-2.327	.108	-2.151	.082
T2 - T1	Tutoring with Booklet	.322	.558	209.033	-.996	1.640	.576	.833
T3 - T1	Tutoring with Booklet	-.346	.679	224.823	-1.949	1.257	-.510	.867
T3 - T2	Tutoring with Booklet	-.668	.673	223.223	-2.257	.921	-.992	.583

Table SM7. Study 2: Linear Model. Means and Dispersions for each Time and Group

		Baseline		Post score	
Group	n	Mean	SD	Mean	SD
Unrelated Topic	20	16.45	3.27	17.15	2.89
Tutoring	30	17.20	3.31	18.87	1.96
Tutoring with Booklet	27	16.52	3.62	19.11	2.06
Expert	20	16.45	2.24	19.70	1.78

Table SM8. Study 2: LM2 Estimates by Each Level of Independent Variable

Coefficients	Estimate	SE	t value	Pr(>|t|)	2.5%	97.5%
Intercept	12.061	1.125	10.721	.000	9.826	14.295
Baseline	.309	.063	4.907	.000	.184	.435
Tutoring	1.485	.565	2.630	.010	.363	2.606
Tutoring with Booklet	1.940	.575	3.374	.001	.798	3.082
Expert	2.550	.616	4.138	.000	1.326	3.774

Table SM9. Study 2: LM2 Fitting Information

R squared	Adjusted. r squared	Sigma	Statistic	p value	df	Log Likelihood	AIC	BIC	Deviance	df residual
.320	.291	1.949	10.836	.000	5	-199.795	411.589	427.037	349.432	92

Table SM10. Study 2: Results of ANOVA (Baseline by Group)

Factor	Sum of Squares	df	F value	Pr(>F)
Baseline	91.451	1	24.078	.000
Group	72.222	3	6.338	.001
Residuals	349.432	92		

Table SM11. Study 2: Descriptive Statistics of Mention and Post Score (Groups T and TB)

Group	Mean mention	SD mention	Post Score Mean	Post Score SD	n
Tutoring	9.643	3.664	18.821	2.019	28
Tutoring with Booklet	10.680	3.739	19.240	2.047	25

Table SM12. Study 2: LM2m Coefficient Estimation

Coefficients	Estimate	SE	t value	Pr(>|t|)	2.5%	97.5%
(Intercept)	14.116	1.608	8.781	.000	10.887	17.345
Baseline	.191	.080	2.387	.021	.030	.352
Mention	.163	.071	2.313	.025	.022	.305

Table SM13. Study 2: LM2m Fitting Information

R squared	Adjusted r squared	Sigma	Statistic	p value	df	Log likelihood	AIC	BIC	Deviance	Df residual
.170	.137	1.88	5.117	.010	3	-107.128	222.257	230.138	176.795	50

Supplemental Material References

1. Roscoe RD, Chi MT, 2007. Understanding tutor learning: Knowledge-building and knowledge-telling in peer tutors' explanations and questions *Rev Educ Res 77*: 534-574.

2. Cohen J, Cohen P, West SG, Aiken LS,2003. Applied Multiple Regression/Correlation Analysis for the Behavioral Sciences (3rd edition), Mahwah: USA: Lawrence Earlbaum Associates.

3. Wickham H, Henry L, 2019. tidyr: Tidy Messy Data. R package version 1.0.0. Available at: https://CRAN. R-project.org/package=tidyr. Accessed December 10, 2020.

4. Wickham H, François R, Henry L, Müller K; 2019. A grammar of data manipulation. R package version 0.8.3. Available at: https://CRAN.R-project.org/package=dplyr Accessed December 10, 2020.

5. Bates D, Mächler M, Bolker B, Walker S, 2014. Fitting linear mixed-effects models using lme4 *arXiv preprint*: *arXiv: 1406.5823*.

6. Fox J, Weisberg S, 2019. An {R} Companion to Applied Regression, Third Edition. California, USA: Sage.

7. Lenth R, 2019. emmeans: estimated marginal means, aka least-squares means. R package v. 1.3. 4. Available at: https://CRAN.R-project.org/package= emmeans. Accessed December 10, 2020.

8. Gohel D, 2019. flextable: Functions for Tabular Reporting. R package version 0.5.5. Available at: https://CRAN.R-project.org/package= flextable. Accessed December 10, 2020.

9. Wickham H, 2016. ggplot2: Elegant Graphics for Data Analysis. New York, USA: Springer-Verlag.

10. AhbiRami R, Zuharah WF, 2020. School-based health education for dengue control in Kelantan, Malaysia: Impact on knowledge, attitude and practice *PLoS Negl Trop Dis14*: e0008075.
